# Potential of Ultraviolet-Visible Spectroscopy for the Differentiation of Spanish Vinegars According to the Geographical Origin and the Prediction of Their Functional Properties

**DOI:** 10.3390/foods10081830

**Published:** 2021-08-07

**Authors:** Raúl González-Domínguez, Ana Sayago, Ángeles Fernández-Recamales

**Affiliations:** 1 AgriFood Laboratory, Faculty of Experimental Sciences, University of Huelva, 21007 Huelva, Spain; ana.sayago@dqcm.uhu.es (A.S.); recamale@dqcm.uhu.es (Á.F.-R.); 2 International Campus of Excellence CeiA3, University of Huelva, 21007 Huelva, Spain

**Keywords:** vinegar, protected designation of origin, UV-Vis spectroscopy, authentication, prediction

## Abstract

High-quality wine vinegars with unique organoleptic characteristics are produced in southern Spain under three Protected Designations of Origin (PDO), namely “Jerez”, “Condado de Huelva” and “Montilla-Moriles”. To guarantee their authenticity and avoid frauds, robust and low-cost analytical methodologies are needed for the quality control and traceability of vinegars. In this study, we propose the use of ultraviolet-visible spectroscopy in combination with multivariate statistical tools to discriminate Spanish wine vinegars according to their geographical origin, as well as to predict their physicochemical and functional properties. Linear discriminant analysis provided a clear clustering of vinegar samples according to the PDO with excellent classification performance (98.6%). Furthermore, partial least squares regression analysis demonstrated that spectral data can serve as accurate predictors of the total phenolic content and antioxidant activity of vinegars. Accordingly, UV-Vis spectroscopy stands out as a suitable analytical tool for simple and rapid authentication and traceability of vinegars.

## 1. Introduction

Vinegar is a condiment widely employed in the Mediterranean and Asian diets to preserve and improve the sensory characteristics of foods. According to the Codex Alimentarius Commission, vinegars can be obtained from different agricultural products rich in starch and/or sugars by means of a double fermentation process (i.e., alcoholic and acetic fermentation). In Europe, grapes, apple, pomegranate, cherry and other fruit juices are the most commonly used raw materials for vinegar production, whereas Asian vinegars are normally based on cereals such as sorghum, rice, sticky rice and others [1]. Spain is one of the major producers of high-quality wine vinegars worldwide, whose production is mainly concentrated in Andalusia (Southern Spain). A singularity of the wines and vinegars produced in this geographical area is the use of the traditional aging system of “criaderas and soleras” [2], which provides them with unique organoleptic characteristics that are highly appreciated by consumers. As a result of these unique characteristics, three Protected Designations of Origin (PDO) of Andalusian vinegars have been recognized in accordance with the European Community legislation (Council Regulation (EC) No 510/2006) [3], namely the PDO “Vinagre de Jerez”, registered in 1995; the PDO “Vinagre del Condado de Huelva”, registered in 2002; and the PDO “Vinagre de Montilla-Moriles”, registered in 2008 [4,5,6].

The composition and organoleptic characteristics of vinegars are influenced by multiple factors, such as the raw material used as a substrate, the acetification system, or the time and method employed for vinegar aging in wooden barrels (e.g., “criaderas and soleras” system, “añada” system), among others [7,8]. These chemical variations, mainly in terms of organic acids and polyphenolic compounds, can in turn affect the functional properties of vinegars (e.g., acidity, antioxidant activity). In this respect, Budak et al. have reported that functional and therapeutic properties of vinegar on human health comprise antibacterial activity, blood pressure reduction, antioxidant activity, prevention of cardiovascular diseases, and improved blood glucose response [9]. Altogether, it becomes evident that the food industry requires robust analytical methodologies to characterize the quality and verify the geographical origin of vinegars. In this context, several studies have previously reported the possibility of discriminating vinegars by using classical targeted approaches based on atomic emission spectroscopy, gas chromatography–mass spectrometry and high-performance liquid chromatography for the determination of mineral elements [10], volatile compounds [11,12,13], as well as polyphenols, organic acids and amino acids [13,14], respectively. Although these methods generally provide high accurateness and sensitivity, they are also time consuming and require considerable amounts of toxic and expensive chemical solvents and reagents. As an alternative, rapid non-targeted spectroscopic methods have been proposed for food quality control and authentication [15]. Among them, near infrared (NIR), mid infrared (MIR), Fourier transform infrared (FTIR), ultraviolet-visible (UV-Vis) and fluorescence spectroscopies, combined with multivariate approaches, have successfully been applied for the characterization and authentication of vinegars [16,17], their quality control [17,18], and the detection of adulterations [7,19].

The main aim of this work was to investigate the potential of UV-Vis spectroscopy in combination with chemometric tools for discriminating Andalusian PDO wine vinegars according to their geographical origin. Secondly, we aimed to predict the physicochemical and functional properties of vinegars using the spectral information by applying regression analysis.

## 2. Materials and Methods

### 2.1. Vinegar Samples

A total of 71 vinegar samples were kindly provided by local wine cellars from the three Andalusian PDOs: 18 “Condado de Huelva” PDO vinegar samples, 8 “Montilla-Moriles” PDO vinegar samples, and 45 “Jerez” PDO vinegar samples. All the samples were kept in darkness at room temperature until analysis.

### 2.2. Spectral Measurements

Absorption spectra in the ultraviolet-visible (UV-Vis) region were recorded in the range 200–700 nm at wavelength intervals of 2 nm with a scanning speed of 2400 nm min^−1^, using a Thermo Electron Corporation Spectronic Helios Alpha spectrophotometer (Thermo Scientific^TM^, Waltham, MA, USA). The absorbance was measured using rectangular quartz cuvettes with a path length of 2 mm against deionized water blanks. 

### 2.3. Determination of Chemical Parameters

#### 2.3.1. Total Phenolic Content

The total phenolic content (TPC) of vinegars was measured according to the Folin–Ciocalteu spectrophotometric method [20]. For this purpose, a volume of 0.02 mL of each sample was mixed with 1.58 mL of Milli-Q water and 0.10 mL of the Folin–Ciocalteu reagent (Sigma-Aldrich, Steinheim, Germany). Then, 0.30 mL of 20% sodium carbonate was added, and the mixture was incubated at 40 °C during 120 min using a water bath. Finally, the absorbance was measured at 725 nm using a quartz cuvette against Milli-Q water blanks. The results were expressed as gallic acid equivalents per liter.

#### 2.3.2. Antioxidant Activity 

The antioxidant activity of vinegar samples was examined using the method developed by Brand-Williams et al. based on the redox reaction between the radical 1,1-diphenyl-2-picrylhydrazyl (DPPH) and the antioxidants contained in the sample [21]. For this, a total volume of 0.1 mL of the vinegar sample was added to 3.9 mL of 0.1 mM DPPH solution in methanol. The mixture was kept at room temperature for 30 min, and the absorbance was then measured at 515 nm. Complementarily, the ABTS (2,2’-azino-bis(3-ethylbenzothiazoline-6-sulfonic acid)) radical cation decolorization assay was also applied to determine the free-radical scavenging activity of vinegar samples according to the method of Pellegrini et al. [22]. To this end, the ABTS radical cation (ABTS^•+^) was first produced by reacting 7 mM ABTS stock solution with 2.45 mM potassium persulfate at room temperature in darkness for 16 h. Then, the ABTS^•+^ solution was diluted with ethanol to obtain an absorbance of 0.70 (±0.01) at 734 nm. Finally, a 0.1 mL aliquot of the sample was added to 2.9 mL of the diluted ABTS^•+^ solution, the mixture was incubated for 6 min, and the absorbance was read at 734 nm. The results of both the DPPH and ABTS assays were expressed as Trolox equivalents per liter.

#### 2.3.3. Total Acidity and pH

Total acidity was measured using a Titralyser automatic titrator (Laboratoires Dujardin-Salleron, Noizay, France) according to the Spanish Official Methods for the analysis of vinegars [23]. The results were expressed as grams of acetic acid per 100 mL of vinegar. The pH measurements were performed using the same automatic titrator.

### 2.4. Data Analysis

Data processing and statistical analyses were performed in the Statistica 8.0 software (StatSoft, Tulsa, OK, USA). To remove undesirable systematic variation in the data due to physical effects, different preprocessing methods were studied prior to multivariate analysis, including standard normal variate (SNV), multiplicative scatter correction (MSC), first derivative (1D) and second derivative (2D). Furthermore, both the raw spectra and the preprocessed spectra were subjected to logarithmic transformation and normalized by autoscaling. Briefly, techniques such as SNV, MSC and autoscaling normalization attempt to reduce spectral scattering, whereas spectral derivatives may help to remove baseline drift, distinguish overlap peaks and extract important signals [24]. Analysis of variance (ANOVA) followed by the Fisher LSD post hoc test was applied to look for differences between the three PDOs under study in terms of the chemical parameters evaluated here (i.e., TPC, antioxidant activity, total acidity, pH). *p*-Values below 0.05 were considered as statistically significant. Afterwards, linear discriminant analysis (LDA) was employed to build classification models with the aim of assessing the potential of spectroscopy data to authenticate wine vinegars according to the geographical origin. LDA is based on the generation of a number of orthogonal linear discriminant functions equal to the number of categories minus one [25]. Prior to LDA, data normality was checked by inspecting probability plots, and the homogeneity of variance-covariance matrices was tested by applying the Box’s M test. Furthermore, the multicollinearity was assessed by using the condition number method, which is defined as the square root of the ratio between the maximum and minimum eigenvalues. The most significant variables involved in sample differentiation according to the PDO were selected using Wilks’ λ and F value as criterion for inclusion or removal of variables in the model. Subsequently, the LDA models were subjected to 7-fold cross-validation to assess their predictive ability. For this purpose, the data matrix was randomly divided into two sets, both of them containing the same percentage of samples within each class: a training set that was used to construct the classification model, and a test set to evaluate the model performance. The performance of the models was evaluated by computing their sensitivity (SENS) and specificity (SPEC), where SENS refers to the percentage of cases belonging to a determinate class that were correctly classified, and SPEC refers to the percentage of cases not belonging to a class that were correctly not classified in this class. Finally, partial least squares regression (PLSR) analysis was applied to predict the chemical and functional properties of vinegars from the spectral data. This technique is a quick, efficient and optimal regression method based on covariance, which is highly recommended to avoid overfitting when the number of explanatory variables is large and inter-correlated. This technique is based on building a set of components that accounts for as much as possible variation in the data, while also modeling the Y variables. For this purpose, PLSR works by extracting a set of components that transforms the original X and Y data into a set of t-scores and u-scores, respectively. Then, the t-scores are used to predict the u-scores, which are in turn used to predict the response variables. The statistical performance of these models can be defined by the following parameters: R^2^_y_, the proportion of variance of the response variable explained by the model; and R^2^_x_, the proportion of variance in the data explained by the model. Furthermore, their predictive ability was assessed by computing the regression correlation coefficient (R^2^), the predicted residual error sum of squares (PRESS) and the residual predictive deviation (RPD). The coefficient of correlation estimates the percentage of variation explained by the model, whereas the PRESS parameter provides a measure of the fit of the regression to a sample of observations that were not used to create the model. The RPD values were computed as the ratio between the standard deviation of the reference values and the error of prediction, so that the higher the RPD values, the greater the probability of the model to accurately predict the chemical parameters. Accordingly, good prediction models require R^2^ values to be close to 1, as small as possible PRESS values, and RPD values above 2.5–3.0 [26].

## 3. Results and Discussion

### 3.1. Spectral and Chemical Characteristics of Vinegars

**Figure 1:**
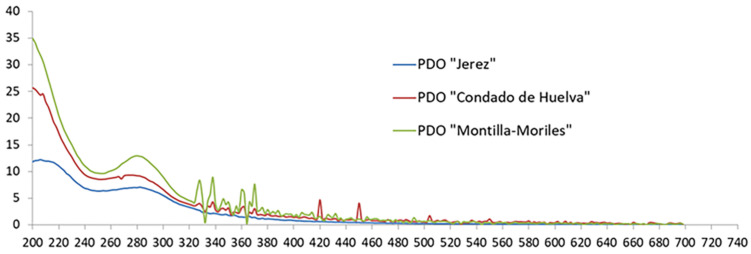
Typical UV-Vis spectra of vinegars.

The spectral data recorded in this study were in accordance with the characteristic UV-Vis spectra of vinegars reported in the literature [7]. In these spectra, three regions can easily be differentiated in all the vinegar samples regardless of the geographical origin (Figure 1): a strong absorption peak around 200 nm (region of sobresaturation), the absorption band of phenolic compounds in the range 250–400 nm, and the region above 400 nm, where there is practically no absorption.

Furthermore, four parameters related to the physicochemical and functional properties of vinegars were also assessed in this study. The Folin–Ciocalteu method was employed for determining the TPC of vinegars, whereas the antioxidant activity was determined by applying two complementary methods, namely the DPPH and ABTS assays [27]. In addition, the total acidity and pH of the vinegar samples were measured using a titrator. As shown in Table 1, the TPCs and antioxidant activities were within the ranges reported by Kadiroğlu for various types of commercial vinegars [19], whereas total acidity values were similar to those found by De la Haba et al. in vinegars from the PDO “Montilla-Moriles” [16]. In contrast, the pH values were slightly lower than those reported by these same authors. One-way ANOVA revealed significant differences in the four parameters determined here between the samples from the three Andalusian PDOs under study (Table 1). Vinegars from the “Montilla-Moriles” PDO showed a characteristic chemical profile with higher TPC and antioxidant activity, whereas “Condado de Huelva” and “Jerez” samples were characterized by higher total acidity.

### 3.2. Differentiation of Vinegars According to Their Protected Designation of Origin

To evaluate the potential of UV-Vis spectroscopy to differentiate vinegar samples according to the geographical origin, the spectral data recorded here were subjected to linear discriminant analysis (LDA). Furthermore, we also compared the potential of different preprocessing methods to correct data variations that can be caused by physical phenomena (e.g., noise, baseline drift), with the aim of improving the classification performance of the multivariate models. 

**Figure 2:**
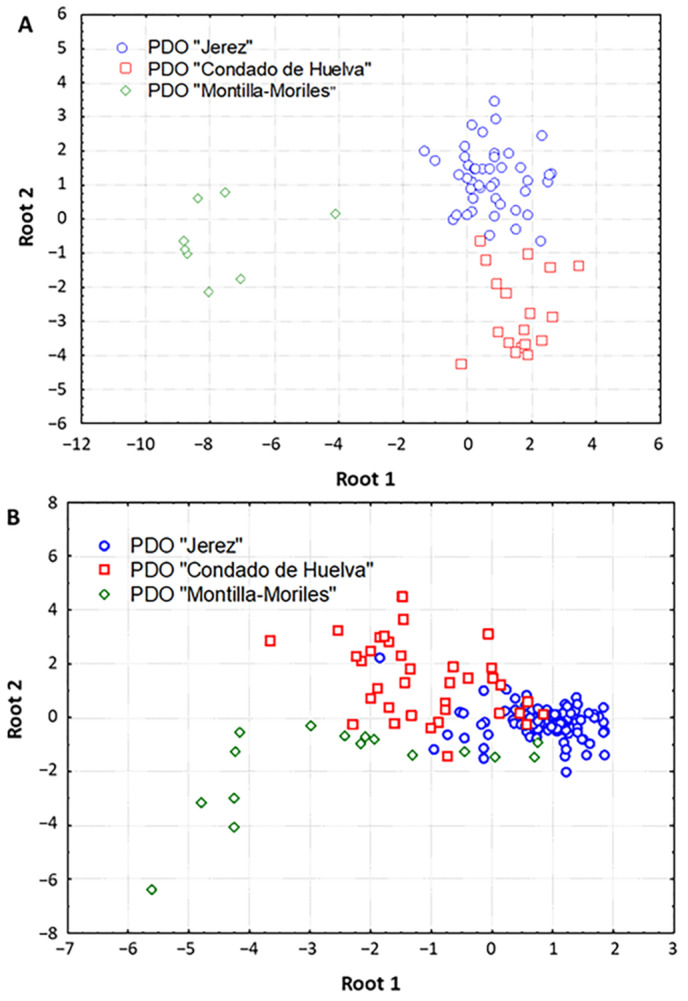
Linear discriminant analysis (LDA) scatter plots showing the distribution of samples in the space defined by the two first canonical variables using: (**A**) non-preprocessed UV-Vis spectral data, (**B**) SNV-preprocessed UV-Vis spectral data.

For stepwise LDA modeling, the original variables were divided into five wavelength intervals, as LDA requires a maximum number of variables equal to the number of cases. Table 2 shows the percentage of correct classifications obtained for each model, the number of components selected, as well as the sensitivity (SENS) and specificity (SPEC) parameters computed by means of cross-validation. The best results were obtained when LDA was carried out on non-preprocessed data in the range 280–400 nm, with 98.6% mean prediction ability (only one “Condado de Huelva” sample was misclassified). This model retained eight components (F to enter = 4.00 and F to remove = 1.00), and the scatter plot of the samples in the plane defined by the two first canonical variables enabled a clear distinction of the vinegar samples according to the PDO. As shown in Figure 2A, the first canonical function differentiated the “Montilla-Moriles” vinegar samples from the other two PDOs, which were in turn separated by the second component. Moreover, the comparison of the different preprocessing techniques demonstrated that SNV-based treatment of the spectral data in the region 502–600 nm also provided good classification performance (88.7%), although the differentiation of the three PDOs in the corresponding scatter plot was not so clear compared to that obtained with non-preprocessed data (Figure 2B). This is in good agreement with previous studies describing the suitability of SNV for the extraction of spectral information related to antioxidants and antioxidant activity by NIR [28], and for the discrimination of Australian Shiraz wines by MIR [29]. 

In a previous study, vinegar samples from “Jerez” and “Condado de Huelva” PDOs were subjected to complete chemical characterization of polyphenols and volatile compounds [13]. Using these chemical descriptors, LDA analysis enabled the differentiation of the samples according to the geographical origin, although yielding lower classification performance to that provided by UV-Vis spectral data (92.86% and 94.12% of the samples were successfully classified when modeling polyphenolic and volatile data, respectively). This therefore highlights the great potential of spectroscopic techniques as simple, ecofriendly and low-cost alternatives against traditional analytical approaches based on chemical analysis for the differentiation of vinegar samples.

### 3.3. Prediction of Chemical Parameters of Vinegars Using Spectroscopic Data

**Figure 3:**
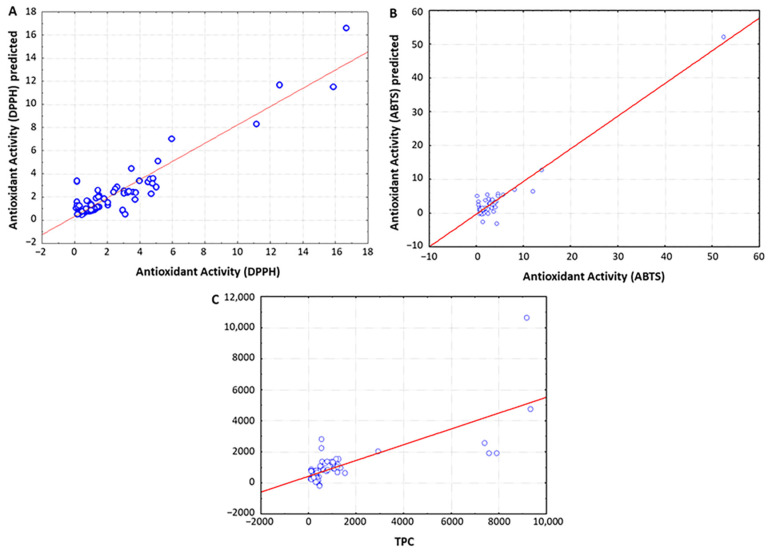
Partial least squares regression (PLSR) analysis for predicting the antioxidant activity measured through the DPPH assay (**A**) and the ABTS assay (**B**), as well as the total phenolic content (**C**) of vinegars using UV-Vis spectral data.

To investigate the potential of spectroscopy techniques for predicting the chemical and functional characteristics of vinegar samples, multivariate partial least squares regression (PLSR) was applied to model the linear relationships between the whole UV-Vis spectral data and the chemical parameters under study (i.e., TPC, antioxidant activity, total acidity and pH). As shown in Table 3 and Figure 3, the spectroscopic data demonstrated excellent capacity to predict the antioxidant activity and TPC of vinegars, as expected considering the strong absorption band of phenolic compounds in the UV-Vis region. The best model for predicting the antioxidant activity measured through the DPPH assay was obtained when using non-preprocessed spectral data, which was constructed with three PLS factors accounting for 91.5% and 84.5% of the variability for X and Y variables, respectively (R^2^ = 0.849, PRESS = 0.561, RPD = 6.95). Similarly, the spectral data also accurately predicted the antioxidant activity determined by means of the ABTS assay, in that case when applying MSC preprocessing (R^2^ = 0.990, PRESS = 0.220, RPD = 13.18). These results are in great accordance with those reported by Kadiroğlu, who used FTIR spectral data to predict the antioxidant activities of commercial vinegars [19]. With regard to the TPC, the best predictive model was also obtained after applying MSC preprocessing to the spectral data (R^2^ = 0.744, PRESS = 0.715, RPD = 2.75), in line with a previous study describing the application of NIR spectroscopy to characterize “Montilla-Moriles” PDO vinegars [16]. In this respect, it should be noted that regression analysis between non-processed spectral data and TPC provided a higher RPD value to that obtained when using MSC-preprocessed data (Table 3), but the R^2^ parameter was below the recommendations by Tamaki and Mazza for accurate predictions [26].

In contrast, PLSR models for total acidity and pH showed poor predictive ability regardless of the preprocessing technique applied, with R^2^ values below 0.4 for both chemical parameters, and RPD values below 1.45 and 0.26 for total acidity and pH, respectively. These results were, however, not surprising since the principal acidity-related compounds present in vinegars do not absorb in the UV-Vis region.

## 4. Conclusions

In this study, we have demonstrated that UV-Vis spectroscopy in combination with chemometrics tools can be used for the authentication of wine vinegars from Andalusian PDOs, namely “Vinagre del Condado de Huelva”, “Vinagre de Montilla-Moriles” and “Vinagre de Jerez”. This novel spectroscopic method represents a simple, ecofriendly and low-cost alternative against traditional analytical approaches based on chemical analysis. In particular, LDA modeling of the spectral data recorded within the range 280–400 nm, where phenolic compounds show their characteristic absorption band, enabled a clear differentiation of the three PDOs under study with excellent classification performance (98.6%). Furthermore, PLS regression analysis demonstrated the capacity of UV-Vis spectral data for predicting the TPC and antioxidant activity of vinegars. Altogether, the present study represents one-step further on the development of fast-screening methods for quality control and traceability of wine vinegars. Future studies involving a higher number of vinegar samples are needed to validate the results and conclusions presented here, thus enabling the implementation of this methodology for routine analysis in vinegar certification.

## Figures and Tables

**Table 1:** Mean, minimum and maximum values for the antioxidant activity, total phenolic content, total acidity and pH in vinegar samples from the three Andalusian PDOs, and *p*-values obtained by ANOVA.

	PDO “Jerez”	PDO “Condado de Huelva”	PDO “Montilla-Moriles”	*p*-Value
Antioxidant activity—DPPH assay (mmol Trolox equivalents L^−1^)	1.41 ^a^ (0.30–5.00)	1.39 ^a^ (0.08–5.94)	5.37 ^b^ (0.69–16.68)	0.0000
Antioxidant activity—ABTS assay (mmol Trolox equivalents L^−1^)	2.11 ^a^ (0.29–4.35)	1.45 ^a^ (0.15–4.53)	15.24 ^b^ (0.87–52.33)	0.0001
Total phenolic content (mg gallic acid equivalents L^−1^)	450.69 ^a^ (157.54–1347.56)	984.26 ^a^ (102.82–9341.47)	2186.14 ^b^ (206.99–7906.01)	0.0000
Total acidity (g acetic acid 100 mL^−1^)	8.44 ^a^ (5.58–10.89)	8.72 ^a^ (6.54–11.04)	7.32 ^b^ (5.88–10.32)	0.0079
pH	2.03 ^a^ (1.85–2.42)	1.95 ^b^ (1.60–2.36)	2.14 ^c^ (1.60–2.57)	0.0029

Superscript letters within each row indicate significant differences between groups marked with different letters, according to the post-hoc Fisher LSD test (*p* < 0.05).

**Table 2:** Statistical performance of the linear discriminant analysis (LDA) models built for the differentiation of vinegars according to the PDO using the UV-Vis spectral data.

Wavelength Interval	Preprocessing Method	Number of Components	Classification Performance	SENS	SPEC
200–278 nm	Raw	4	81.0%	66.0%	83.5%
	SNV	2	76.0%	56.6%	81.6%
	MSC	2	77.0%	60.3%	82.9%
	1D	4	77.0%	58.2%	83.7%
	2D	7	77.5%	45.6%	74.5%
280–400 nm	Raw	8	98.6%	77.8%	89.4%
	SNV	8	83.8%	59.6%	84.7%
	MSC	9	83.0%	61.1%	85.7%
	1D	7	78.0%	44.8%	74.1%
	2D	3	68.3%	43.5%	70.8%
402–500 nm	Raw	3	69.0%	45.5%	73.9%
	SNV	7	82.3%	62.0%	84.0%
	MSC	7	80.0%	59.1%	84.0%
	1D	3	68.0%	54.4%	77.7%
	2D	6	71.8%	37.5%	70.6%
502–600 nm	Raw	3	71.0%	48.3%	75.4%
	SNV	10	88.7%	63.7%	85.3%
	MSC	11	85.9%	61.7%	84.6%
	1D	5	71.8%	42.5%	69.9%
	2D	4	67.6%	42.2%	70.9%
602–698 nm	Raw	11	81.7%	45.0%	74.1%
	SNV	2	80.0%	59.2%	82.4%
	MSC	5	80.0%	60.7%	83.8%
	1D	2	67.6%	46.3%	72.5%
	2D	2	66.9%	41.3%	70.0%

**Table 3:** Statistical performance of the partial least squares regression (PLSR) models built for the prediction of the chemical properties of vinegars using the UV-Vis spectral data.

Wavelength Interval	Preprocessing Method	Number of Components	R^2^_Y_	R^2^_X_	R^2^	PRESS	RPD
Antioxidant activity (DPPH assay)	Raw	3	0.845	0.915	0.849	0.561	6.95
	SNV	5	0.469	0.918	0.815	0.773	5.04
	MSC	5	0.894	0.906	0.870	0.703	5.54
	1D	1	0.727	0.318	0.818	1.132	3.44
	2D	9	0.970	0.809	0.845	0.582	6.69
Antioxidant activity (ABTS assay)	Raw	8	0.992	0.973	0.983	0.306	9.50
	SNV	6	0.969	0.909	0.985	0.455	6.37
	MSC	2	0.878	0.838	0.990	0.220	13.18
	1D	1	0.944	0.625	0.945	0.266	10.91
	2D	1	0.953	0.535	0.951	0.236	12.26
Total phenolic content	Raw	2	0.459	0.885	0.456	0.563	3.45
	SNV	2	0.528	0.755	0.659	0.709	2.78
	MSC	2	0.527	0.769	0.744	0.715	2.75
	1D	1	0.516	0.447	0.526	0.687	2.87
	2D	2	0.605	0.559	0.490	0.882	2.23
Total acidity	Raw	1	0.019	0.665	0.053	1.070	1.41
	SNV	6	0.483	0.921	0.219	1.031	1.15
	MSC	6	0.507	0.917	0.394	1.040	1.45
	1D	2	0.109	0.639	0.067	1.038	1.45
	2D	1	0.117	0.189	0.084	1.098	1.37
pH	Raw	4	0.214	0.943	0.074	0.709	0.24
	SNV	4	0.274	0.877	0.183	0.642	0.26
	MSC	6	0.505	0.924	0.384	0.659	0.26
	1D	2	0.111	0.671	0.070	0.683	0.25
	2D	3	0.415	0.605	0.071	0.676	0.25

## Data Availability

The data presented in this study are available on request from the corresponding author.
